# Edible insect biodiversity and anthropo-entomophagy practices in Kalehe and Idjwi territories, D.R. Congo

**DOI:** 10.1186/s13002-022-00575-z

**Published:** 2023-01-05

**Authors:** Jackson Ishara, Marcellin C. Cokola, Ariel Buzera, Mercy Mmari, David Bugeme, Saliou Niassy, Karume Katcho, John Kinyuru

**Affiliations:** 1 Department of Food Science and Technology, Université Evangélique en Afrique, P.O. Box 3323, Bukavu, Democratic Republic of the Congo; 2 Department of Food Science and Technology, Jomo Kenyatta University of Agriculture and Technology, P.O. Box 62000-00200, Nairobi, Kenya; 3 Department of Crop Sciences, Université Evangélique en Afrique, P.O Box: 3323, Bukavu, Democratic Republic of the Congo; 4 Department of Biological and Food Sciences, The Open University of Tanzania, P.O. BOX 23409, Dar es Salaam, Tanzania; 5 Crop Production and Protection Unit, Université Catholique de Bukavu, Bukavu, Democratic Republic of the Congo; 6 International Centre of Insect Physiology and Ecology (ICIPE), P.O. Box 30772-00100, Nairobi, Kenya; 7 Functional and Evolutionary Entomology, Gembloux Agro-Bio Tech, University of Liege, Passage des Déportés 2, 5030 Gembloux, Belgium

**Keywords:** Edible insects, Biodiversity, Anthropo-entomophagy, Seasonal availability, Host plants, Harvesting techniques, Processing methods

## Abstract

**Background:**

Located in the Eastern Democratic Republic of Congo (South-Kivu), Kalehe and Idjwi are two relatively unexplored territories with little to no research on edible insects even though anthropo-entomophagy practice is widespread. This study therefore aimed at exploring the biodiversity, perception, consumption, availability, host plants, harvesting techniques, and processing techniques of edible insects.

**Methods:**

Data were collected through a field survey using three techniques, namely structured interviews, direct observations, and insect collection and taxonomy. A total of 260 respondents, 130 in each territory, were interviewed. The field survey focused on inventorying commonly edible insects as well as recording consumer preferences, preference factors, seasonal availability, host plants, harvesting techniques, and processing and preservation methods. Samples for taxonomic characterization were preserved in 70% alcohol.

**Results:**

Nine edible insects, namely *Ruspolia differens* Serville 1838, *Gryllotalpa Africana* Palisot de Beauvois 1805*, Locusta migratoria* Linnaeus 1758, *Macrotermes subhyalinus* Rambur 1842, *Gnathocera trivittata* Swederus 1787, *Rhynchophorus phoenicis* Fabricius 1801, *Vespula spp.* Linnaeus 1758, *Apis mellifera* Linnaeus 1758, and *Imbrasia oyemensis* Rougeot 1955, were recorded as being consumed either as larvae, pupae, and adults. *Ruspolia differens* and *M. subhyalinus* were reported as the most preferred by consumers in the studied territories. A scatter plot of matrices and Pearson's correlations showed a negative correlation between preference based on taste, size, and shape, as well as perceived nutritional value. Their seasonal availability differs from one species to another and correlated with host plants availability. Harvesting techniques and processing and preservation methods depend on species, local knowledge, and practices.

**Conclusion:**

The huge edible insect diversity observed in Kalehe and Idjwi is evidence of anthropo-entomophagy practices in the area. In addition to being an important delicacy and traditional foods, edible insects can contribute to food, environmental, and financial security through local business opportunities. Households can rely on edible insects to meet their nutritional needs instead of conventional livestock. Indigenous practices and technologies used for harvesting, processing, and preserving edible insects must be improved to meet international standards to increase the market and capitalize on the economic potential of edible insects.

## Background

As a result of environmental pressures and global population growth [1], as well as increasing alternative protein demand, edible insects are seen as one of the best options to address global food insecurity [2, 3], due to their nutritional value [4], taste [5], economic [6, 7], and environmental benefits [8]. Among the most consumed edible insect groups, we find a diversity of beetles (Coleoptera, 31%), caterpillars (Lepidoptera, 18%), and bees, wasps, and ants (Hymenoptera, 14%), followed by grasshoppers, locusts, and crickets (Orthoptera, 13%), cicadas, leafhoppers, planthoppers, and bugs (Hemiptera, 10%), termites (blattodea, 3%), dragonflies (Odonata, 3%), flies (Diptera, 2%), and 5% of other orders [9, 10].

These edible insects are generally consumed as eggs, larvae, pupae, adults, or nymphs [11, 12], and most of them are collected from nature [13]. Several species including *Imbrasia oyemensis* (caterpillar) are consumed as both larvae and pupae [11], and *Apis millifera* (honey bee) as eggs, larvae, and pupae [12]. According to Kelemu [14], Lepidoptera (caterpillars) and Hymenoptera are consumed as adults and larvae, while the orders Orthoptera and Hemiptera are consumed as adults. Consumers preference is mainly influenced by familiarity [9, 15], culture [16], palatability [17], and availability as well as local knowledge and processing [15]. People who have eaten them in the past are willing to eat them again, while people to whom such insects are unfamiliar are more likely to avoid eating these insects [18]. Sensory characteristics, nutritional value, customs, and ethnic preferences also play a crucial role in the rate of consumption of insects [9, 15, 19, 20].

The geographical distribution of edible insects’ host plants and seasonality have a major influence on their availability [17], with alate termites, crickets, and caterpillars being more available during the rainy season [21]. The rainy season is generally characterized by abundance of host plants that provide habitat and/or food source for most edible insects [22]. A variety of techniques are used to harvest edible insects, based on species and local knowledge [23]. The most commonly used are handpicking and light trapping [21, 24]. Depending on needs and species, edible insects in Africa are prepared as follows: boiled, fried, dry-fried, stewed, roasted, sun-dried, steamed, salt-roasted, and sometimes eaten raw [21, 25–27].

Anthropo-entomophagy practices have been increasingly documented worldwide [9, 14, 28–35] but less so in the Democratic Republic of Congo (DRC) despite its wide edible insect diversity. A recent study conducted in South-Kivu, in the Fizi, Kabare, Mwenga, and Walungu territories, recorded 23 edible insects [11], but due to ecological, cultural, and dietary habit differences, the results from these territories cannot be extrapolated to Kalehe and Idjwi consumers. There is a need for in-depth research on the diversity of edible insects, the factor influencing their preferences, their seasonal availability, harvesting techniques, and processing techniques for sustainable use, as understanding edible insects' diversity and value chain is critical in promoting edible insects, especially in a context of climate change and ecosystem deforestation, which is currently affecting DRC. This study therefore aimed at exploring the biodiversity, perception, consumption, availability, host plants, harvesting techniques, and processing techniques of edible insects.

## Material and methods

### Study area

**Fig. 1:**
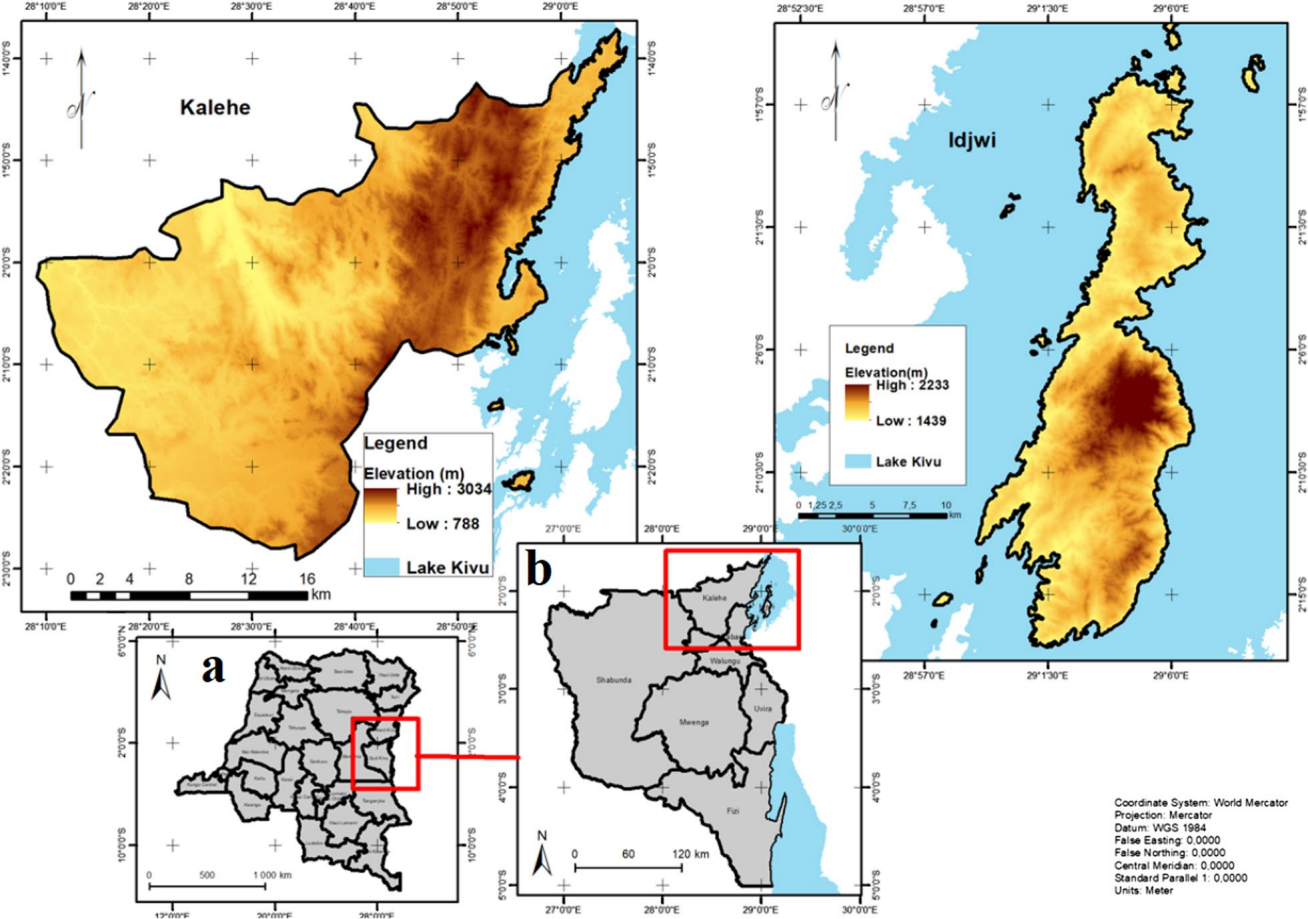
Map showing the Democratic Republic of the Congo (**a**), as well as the South-Kivu Province (**b**), and the study area

Data on diversity, host plants, seasonal availability, harvesting techniques as well as local processing methods, consumption, and preference of edible insects were collected through a survey and direct observations carried out in Kalehe and Idjwi territories in South-Kivu Province, D.R. Congo (Fig. 1). The territories were purposely selected for their familiarity with entomophagy practices and unique agroecological conditions with cultural and dietary habit differences from other territories in DRC, thus influencing edible insects’ availability and preference.

### Agroecological conditions of the study area

The agroecological conditions of the study area are presented in Table 1. The Idjwi Island is located and surrounded by Lake Kivu, located between 1°37′8.85″S and 2°29′5.82″S as well as 29°5′24.23″E and 28°34′15.91″E, with an altitude varying from ~1.439 m to 2.233 m and temperature varying from 17 to 30 °C. As a result of its location (surrounded by a lake) and topography, the climate in Idjwi is humid wet tropical and tropical savannah with a rainfall of 1.500mm each year.

**Table 1:** Agroecological conditions of the study area (CAID and Inogwabini [36])

Characteristics	Territory	
	Kalehe	Idjwi
Area (km [2])	4.197	280.45
Climate type	Humid wet tropical	Humid wet tropical
Dominant soil unity	Haplic Acrisols, Dystric Cambisols and Haplic Nitisols, Humid Ferralsols	Gleyic Solonchaks and Humid Ferralsols
Mean T (°C)	18–22 °C	17–30 °C
Mean annual P(mm)	1.300–2.000	1.540
Estimated population (2019)	933.181	320.009
Density of population (hab km^−2^)	184,6	1.032,3
AEZ*	Low, medium to high	Medium to high

*P* (mm) Precipitation (rainfall); *AEZ* Agroecological zone (high, medium, low); *CAID* Cellule d'Analyses des Indicateurs de Développement (Development Indicators Analysis Unit).

There are two seasons, the dry season (May to August) and the rainy season (September to May), with the dominant soil unities according to WRB (World Reference Base for Soil) as following: Gleyic Solonchaks and Humid Ferralsols, rich in sand and clay. Its vegetation is threatened and naturally shrubby as well as grassy, interspersed with secondary forests. The island is also covered by croplands dominated by coffee, banana, and cassava among others. The Idjwi Territory is among the most densely populated territories in DRC, leading to high pressure on ecosystems.

Located in the North, Kalehe is one of contrasting territory in South-Kivu based on its topography dominated by mountain (the Mitumba) in the East. On the other hand, Kalehe is a bordering territory between South-Kivu and North-Kivu Provinces. Lake Kivu borders Kalehe Territory over a distance of ~86 km from north to south, opening onto the Bukavu basin. The Kalehe Territory is characterized with a humid wet tropical climate and in some areas temperate with altitude. There are two seasons, the rainy season (September to May) and the dry season (June to August), with a precipitation ranging from 1.300 to 2.000 mm each year, and an annual temperature varying between 18 and 22 °C.

A diversity of soil is observed in the Kalehe Territory, from Haplic Acrisols, Dystric Cambisols, Haplic Nitisols, and Humid Ferralsols. The Dystric Cambisols and Haplic Nitisols are rich in clay, very appropriate for agricultural purposes. Its vegetation is dominated by forest, where bamboos and shrubs are unfortunately in the process of disappearing due to an intense deforestation resulting in scarcity of arable land and no appropriate exploitation. Some tea, coffee, banana, and cassava crops on exploited lands are also observed. Other human activities such as small-scale mining, sand mining, and livestock are dominant activities in this area.

### Cultural particularities of the study area

Some cultural particularities of the study area are provided (Table 2). In some Kalehe tribes, especially the Bashi and Bahavu of the third age, there are dietary prohibitions such as the consumption of eggs by young girls and the consumption of chicken meat and cow’s milk by certain categories of people, notably married women, while pregnant women are not allowed to consume chicken eggs, under the pretext that they could give birth to babies without hair. This practice is disappearing among the new generations. Culturally, the Bahavu have a passive resistance, conservatism, and sometimes deviousness. A woman is considered as a source of wealth and does the household chores.

**Table 2:** Cultural particularities of the study area (retrieved from CAID)

Milestone	Territory	
	Kalehe	Idjwi
Tribes	Bahavu (40%) Batembo (25%) Bahunde (1O%) Banyarwanda: Hutu and Tutsi (10%) Bashi, Banyanga, Barega (15%)	Bahavu (95%) Few Pygmies and Rwandans
Spoken languages	Swahili (90%) Kihavu (70%) Kitembo (30%) Kinyarwanda (10%) Kihunde (3%) Mashi (2%)	Kihavu (98%) Swahili (95%)
Main activities	Agriculture 50% Livestock 15% Fishing 15% Artisanal mining 10% Small business and handicrafts 10%	Agriculture (90%) Small business (5%) Fishing (3%) Livestock (2%)
Main agricultural products	Cassava (40%) Potato (25%) Banana (15%) Maize (10%) Beans (10%)	Cassava (55%) Beans (20%). Coffee (15%) Pineapple (10%).
Main non-agricultural products	Honey Mushrooms Caterpillars Fish Cattle, Sheep, Goats Poultry Guinea pigs Wood (firewood, planks, embers) Minerals (gold, cassiterite, coltan, Traumaline, etc.)	Seafood (97%) Sand (3%)
Main source of energy	Wood Petroleum Flashlights Solar Electricity	Wood (50%) Solar (40%) Petroleum (10%)

As for the spoken language (Table 2), Swahili dominates over the local languages, an intercultural language of contact throughout the Territory but spoken by the upper class which are often in contact with non-natives (visitors), but also by merchants and travelers. Kihavu is the mother tongue spoken by the majority of the population in Kalehe Territory (70%), while Kinyarwanda (10%) is the language spoken by a part of Kalehe Territory (the highlands) that is inhabited by Rwandophones (Tutsi and Hutu).

Agriculture is the main activity in Kalehe providing 75% of livelihoods income, which is unfortunately affected by the production decline due to diseases and the population does not have access to seeds resistant to diseases. Before 1996, livestock production was prosperous, but it is now primarily affected by insecurity and repeated wars.

As for Idjwi, this territory has only one large tribe, Bahavus (95%), and a few pygmies as well as Rwandans living there. They are grouped into two sovereign kingdoms headed by a Mwami (at the chiefdom level). Almost all of the population practice subsistence farming.

Kihavu is the vernacular language of Idjwi, while Swahili is the language of contact between the indigenous and urban populations. Cassava, beans, pineapple, and coffee are the main agricultural products. There is a large plantation that occupies almost a third of the arable land in Idjwi Territory, owned by a family that specializes in industrial crops. The breeding of small livestock and poultry are more practiced there, as large livestock is being abandoned due to lack of pasture. Given the population growth and the scarcity of cultivable land, the former pastures have been transformed into food crop fields. Cassava is the flagship product of Idjwi and is therefore the economic booster of this territory. It is produced in all two chiefdoms and in the six groups of the island.

### Sampling and selection of respondents

A total of 260 respondents, 130 respondents in each territory, were interviewed, with priority given to people familiar with entomophagy based on the main objectives. Therefore, the respondents included adults, women and men over 18 years old, from all social classes. A structured oral interview was used individually to ensure better information and minimize external influences on the respondent's side.

### Sources of collected data

**Fig. 2:**
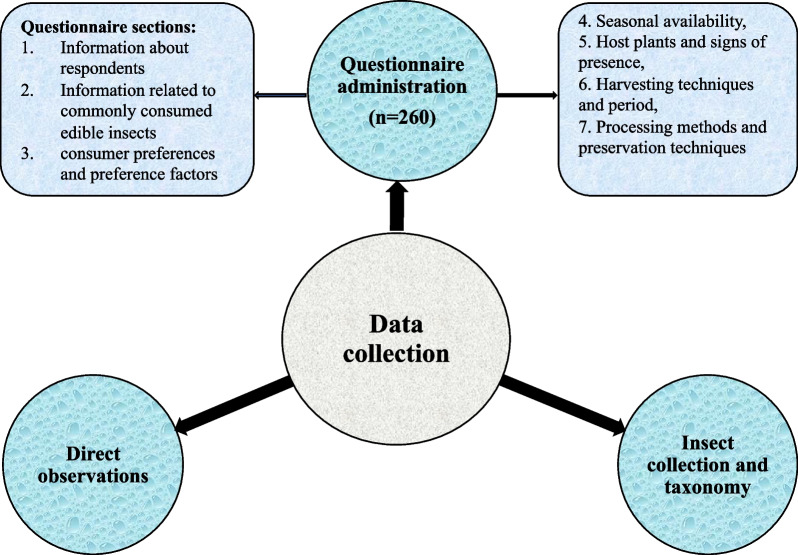
Data collection techniques used to record practices of anthropo-entomophagy in the study area

Data were collected through a field survey using three techniques, namely questionnaire administration, direct observations, insect collection and taxonomy, as depicted in Fig. 2.

#### Questionnaire administration

Structured questionnaires were divided into seven sections to obtain information on local entomophagy knowledge and practices in Kalehe and Idjwi territories. Information about the respondents was collected in the first section. The second section addressed open-ended questions about commonly consumed edible insects, focusing on local names and consumption stages. The third section consisted of collecting information related to preferred edible insects and factors influencing preference, namely perceived nutritional value, color, and shape. In the fourth and fifth sections, questions related to seasonal availability, host plants, and signs of presence were asked, followed by personal observations. The sixth and last sections dealt with harvesting techniques, periods, processing methods, and preservation techniques. The questions were translated into the local dialect to ensure a good understanding for the respondents. Photographs and actual samples of various edible insects identified in the literature were also used to help respondents identify the mentioned insects. Finally, the interviewers clarified some answers to deepen the information sought.

#### Direct observations

Direct observations of relevant information related to insects and their habitats in the study area were recorded from the field. In order to verify and support the answers obtained from the interviewees, pictures were taken. The researchers also took the opportunity to observe how certain edible insects were prepared and consumed.

#### Collection and taxonomic identification of insect samples

The collected samples were preserved in 70% alcohol before being taken to the laboratory at Lwiro Research Center in South-Kivu for identification. A mixture of primary data and taxonomic characters was used to identify and classify the different species of edible insects sampled and collected from the two territories as part of the survey. The taxonomic characters were derived mainly from archival sources and published literature [37]. Then, names of genera and species were obtained by comparison of the morphological characters for each taxon [38] (https://animaldiversity.org/).

### Data analysis

Collected data were analyzed using Microsoft Excel 16.56 and RStudio version 4.2.0. Cleaning of completed questionnaires and verification of information took place. Descriptive and exploratory approaches were used to delineate and describe the existence and use of edible insects in the study area based on the nature of the research questions.

## Results

### Commonly consumed edible insects in selected territories of South-Kivu

**Fig. 3:**
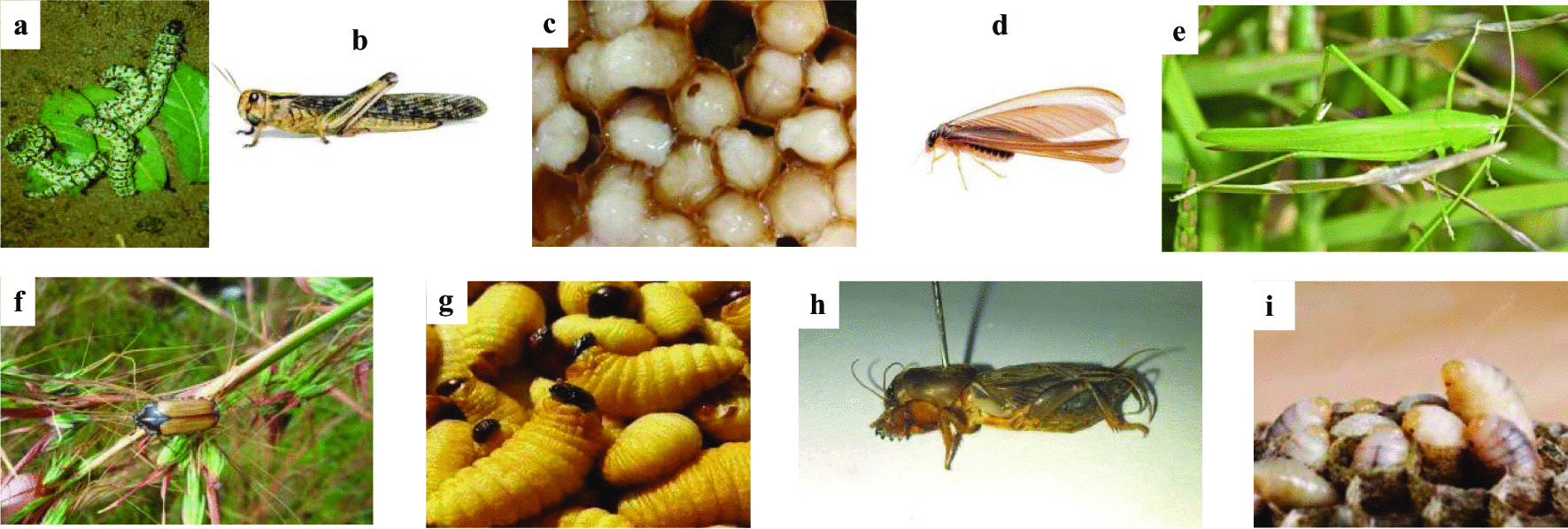
**a** *I. oyemensis* (Caterpillar); **b** *L. migratoria* (Migratory locust); **c** *A. mellifera* larvae (Honey bee); **d** *M. subhyalinus* (Termite); **e** *R. differens* (Grasshopper); **f** *G. trivittata* (Nsike); **g** *R. phoenicis* larvae; **h** *G. africana* (Mole cricket); **i** *Vespula spp.* larvae (Wasp)

Nine edible insects were identified in Kalehe and Idjwi, namely *Ruspolia differens*, *Gryllotalpa Africana, Locusta migratoria*, *Macrotermes subhyalinus*, *Gnathocera trivittata*, *Rhynchophorus phoenicis*, *Vespula spp*., *Apis mellifera*, and *Imbrasia oyemensis* (Table 3). Most of the recorded edible insects are used as food sources in both Kalehe and Idjwi territories (*I. oyemensis*, *L. migratoria*, *A. mellifera*, *M. subhyalinus*, and *R. differens*), while *G. trivittata*, *R. phoenicis*, and *Vespula spp*. are only used in Idjwi, and *G. africana* is only used in Kalehe (Fig. 3). Some of the insects are consumed as larvae including *I. oyemensis*, *A. mellifera*, *R. phoenicis*, and *Vespula spp*., while others are consumed as adults such as *L. migratoria*, *M. subhyalinus*, *R. differens*, *G. trivittata*, and *G. africana*. Unlike others, *I. oyemensis* is consumed as both larvae and pupae, while *A. millifera* is consumed as egg, larvae, and pupae. Honey is also prized much for consumption and commerce.

**Table 3:** Inventoried edible insects in Kalehe and Idjwi territories, Republic Democratic of Congo

Order	Family	Common name	Scientific name	Territory		Local name	Stage of consumption
				Kalehe	Idjwi		
Orthoptera	Tettigoniidae	Grasshopper	*Ruspolia differens* Serville 1838	+	+	Misanani (Idjwi)/Miguku (Kalehe)	Adult
	Gryllotalpidae	Mole cricket	*Gryllotalpa africana*	+	−	Nkwananzi (Kalehe)	Adult
	Acrididae	Migratory locust	*Locusta migratoria* Linnaeus 1758	+	+	Mikelele/Ihanzi (Kalehe)/Panzi (Idjwi & Kalehe)	Adult
Blattodea	Termitidae	Alates termite	*Macrotermes subhyalinus* Rambur 1842	+	+	Iswa (Idjwi/Kalehe); Muchocholi (Kalehe)	Winged adult
Coleoptera	Scarabaeidae	NA	*Gnathocera trivittata* Swederus 1787	−	+	Nsike (Idjwi)	Adult
	Dryophthoridae	Palm weevil	*Rhynchophorus phoenicis* Fabricius 1801	−	+	Bihombogolo (Idjwi)	Larvae
Hymenoptera	Vespidae	Wasp	*Vespula spp.* Linnaeus 1758	−	+	Nsimbwe (Idjwi)	Larvae
	Apidae	Honey bee	*Apis mellifera* Linnaeus 1758	+	+	Binyangu (Idjwi)/Lwasso (Kalehe)	Larvae & pupae
Lepidoptera	Saturniidae	Caterpilar	*Imbrasia oyemensis* Rougeot 1955	+	+	Madaku (Idjwi)	Larvae & pupae

+ : Insects present and consumed

− : Insects not present

*NA* Not applicable (no common name has yet been provided for this species).

All the insects have local names derived from the *Kihavu* and *Kitemba* dialects referring to physical characteristics or uses. Among them, we found Madaku for *I. oyemensis*, *L. migratoria* (Mikelele, Ihanzi, and Panzi), *A. mellifera* (Binyangu and Lwasso), *M. subhyalinus* (Iswa and Muchocholi), *R. differens* (Misanani and Miguku), *G. trivittata* (Nsike), *R. phoenicis* (Bihombogolo), and *G. africana* (Nkwananzi) and Nsimbwe for *Vespula spp*.

### Preference for inventoried edible insects

**Fig. 4:**
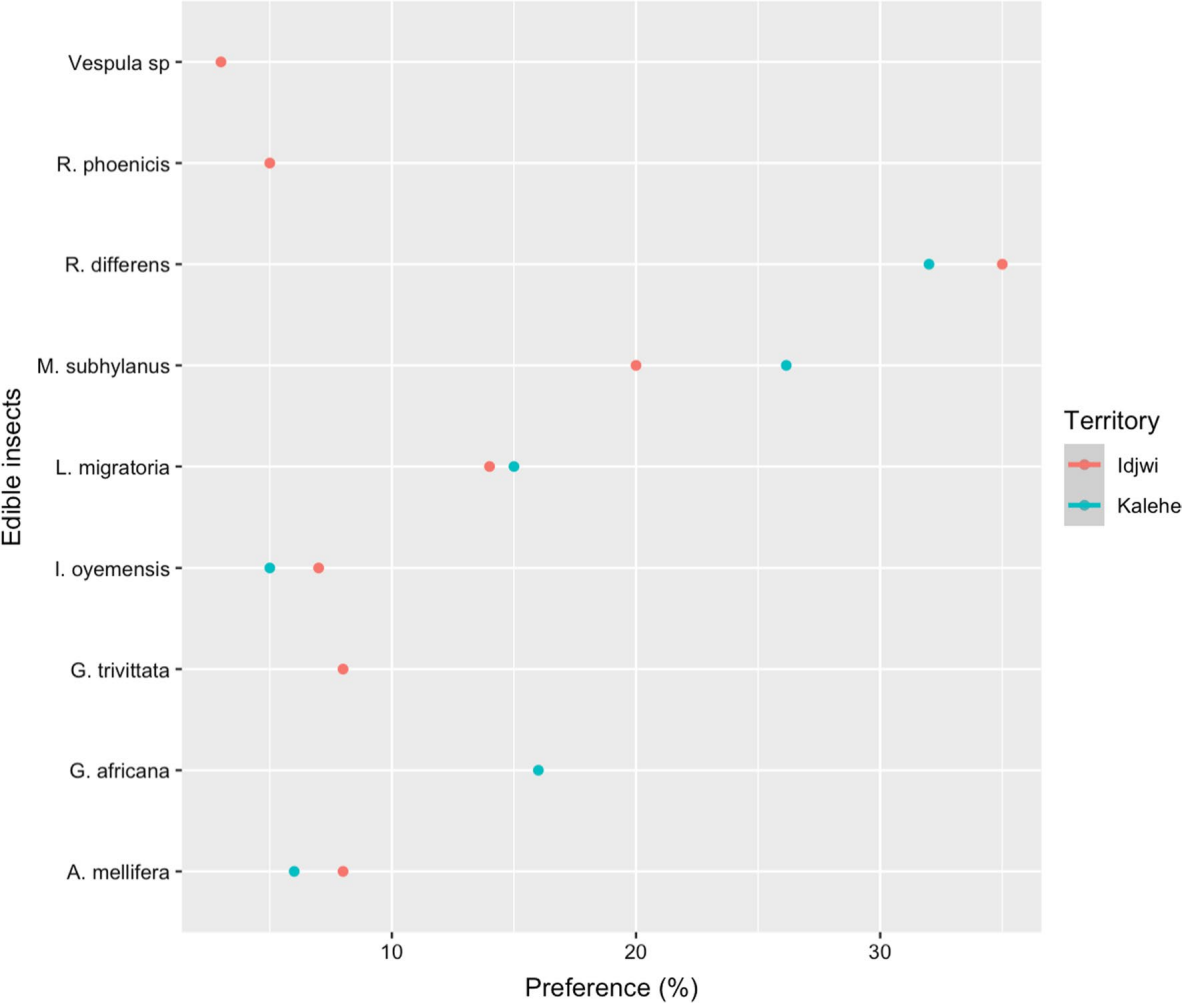
Edible insects’ preference (n = 260, about 130 respondents in each territory)

*Ruspolia differens* and *M. subhyalinus* were the most preferred edible insects in both territories (Fig. 4). In Kalehe, *R. differens* was the most preferred by respondents (32%) followed by *M. subhyalinus* (26%), *G. africana* (16%), *L. migratoria* (15%), *A. mellifera* (6%), and *I. oyemensis* which were the least preferred (5%). In Idjwi, *R. differens* was the most preferred by respondents (35%) followed by *M. subhyalinus* (20%), *L. migratoria* (14%), and *I. oyemensis* (7%), with *R. phoenicis* (5%) and *Vespula spp*. (3%) being the least preferred after *A. mellifera* and *G. trivittata* (8%).

### Factors influencing preference

**Fig. 5:**
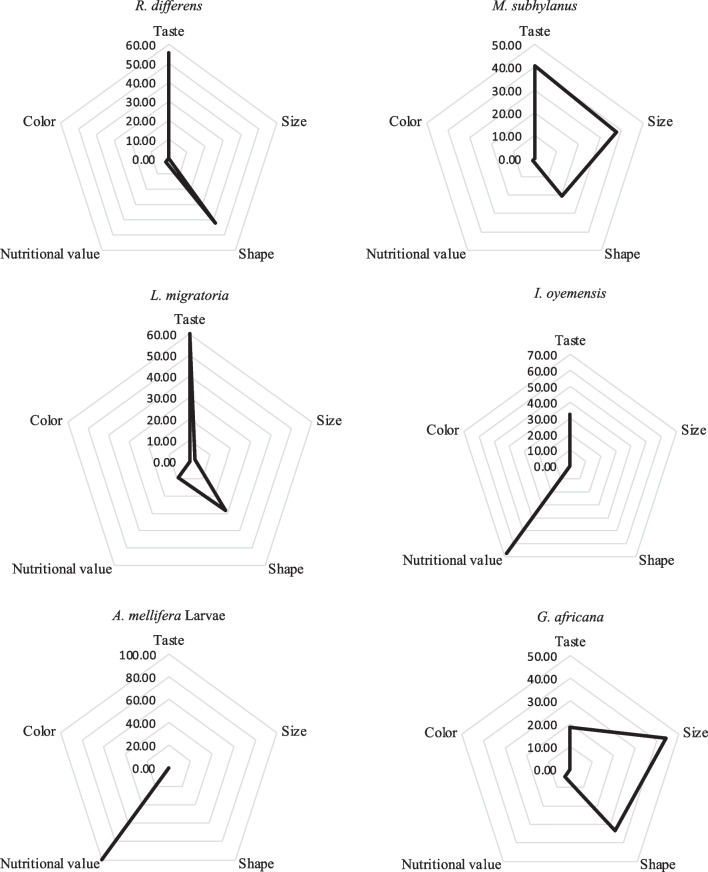
Factors of preference of edible insects in Kalehe Territory (n = 130). Respondents gave reasons for preferring one edible insect over another. The preference was based on taste, size, shape, perceived nutritional value and color

**Fig. 6:**
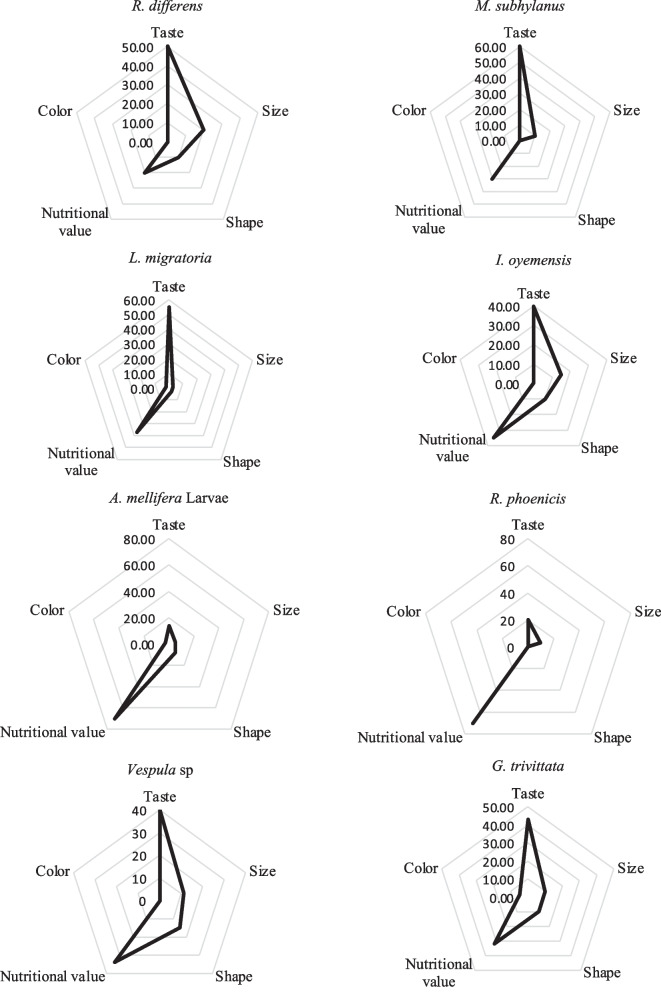
Factors of preference of edible insects in Idjwi Territory (n = 130)

The preference for various edible insects inventoried in the Kalehe and Idjwi territories was found to be influenced by several of factors including taste, shape, size, perceived nutritional value and their colors as plotted in Figs. 5 and 6. Most of them were appreciated for their taste (*R. differens*, *M. subhyalinus*, *L. migratoria*, and *G. trivittata*) and nutritional value (*A. mellifera*, *I. oyemensis*, and *R. phoenicis*).

In Kalehe Territory, *G. africana* was appreciated (18%) for its taste, for its size (44%), for its shape (34%), and for its perceived nutritional value (4%). *I. oyemensis* was appreciated for its taste (32%) and perceived nutritional value (68%). *L. migratoria* was appreciated by 60%, 3%, 28%, and 9% for its taste, size, shape, and nutritional value, respectively; *A. mellifera* larvae were only appreciated for their perceived nutritional value. *Macrotermes subhyalinus* was, respectively, appreciated by 41%, 38%, 20%, and 1% for taste, size, shape, and nutritional value, while *R. differens* was more appreciated for its taste (56%) and moderately appreciated for its shape (42%). *G. africana* was appreciated by 18%, 44%, 34%, and 4% for taste, size, shape, and nutritional value, respectively. No edible insects inventoried were appreciated for their color in Kalehe.

In Idjwi Territory, it was revealed that taste and nutritional value were the main factors of preference (n=130) for edible insects (Fig. 4). *M. subhyalinus* (60%), *L. migratoria* (55%), *R. differens* (50%), *G. trivittata* (43%), *I. oyemensis* (40%), and *Vespula spp*. (40%) were the most appreciated for their taste, while *R. phoenicis* (70%) and *A. mellifera* larvae (60%) were the most appreciated for their nutritional values. In contrast to Kalehe Territory where no edible insects were appreciated for their color, some edible insects were slightly appreciated in Idjwi Territory including *G. trivittata* (5%), *A. mellifera* larvae (3%), and *L. migratoria* (2%).

**Fig. 7:**
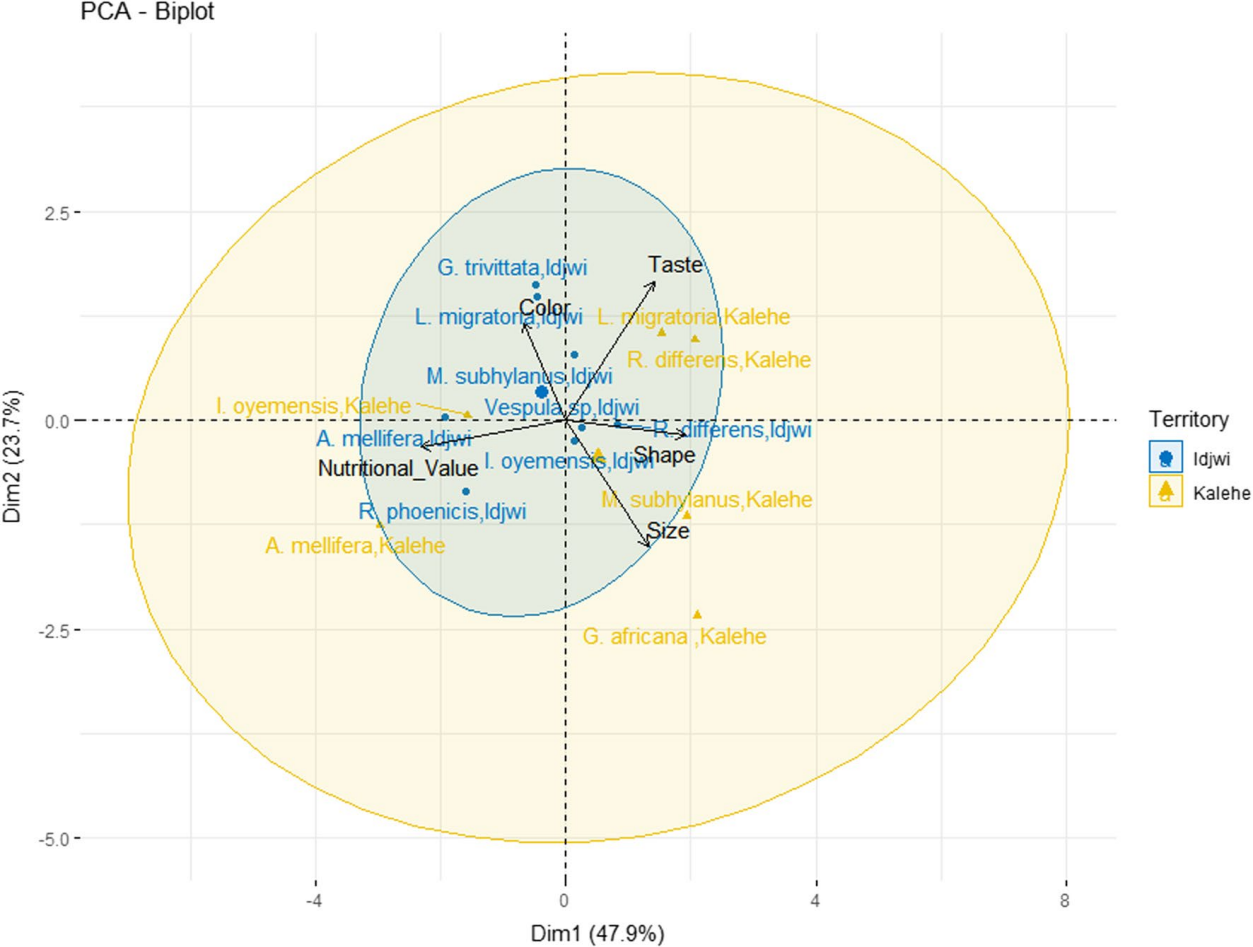
PCA biplot of preference factors

Principal component analysis (PCA biplot) results indicated that the two axes accounted for up to 71.64% of the observed variability in the preference of edible insects in Kalehe and Idjwi based on the preference factors (Fig. 7). The first and second axes accounted for 47.95% and 23.69% of variability, respectively.

**Fig. 8:**
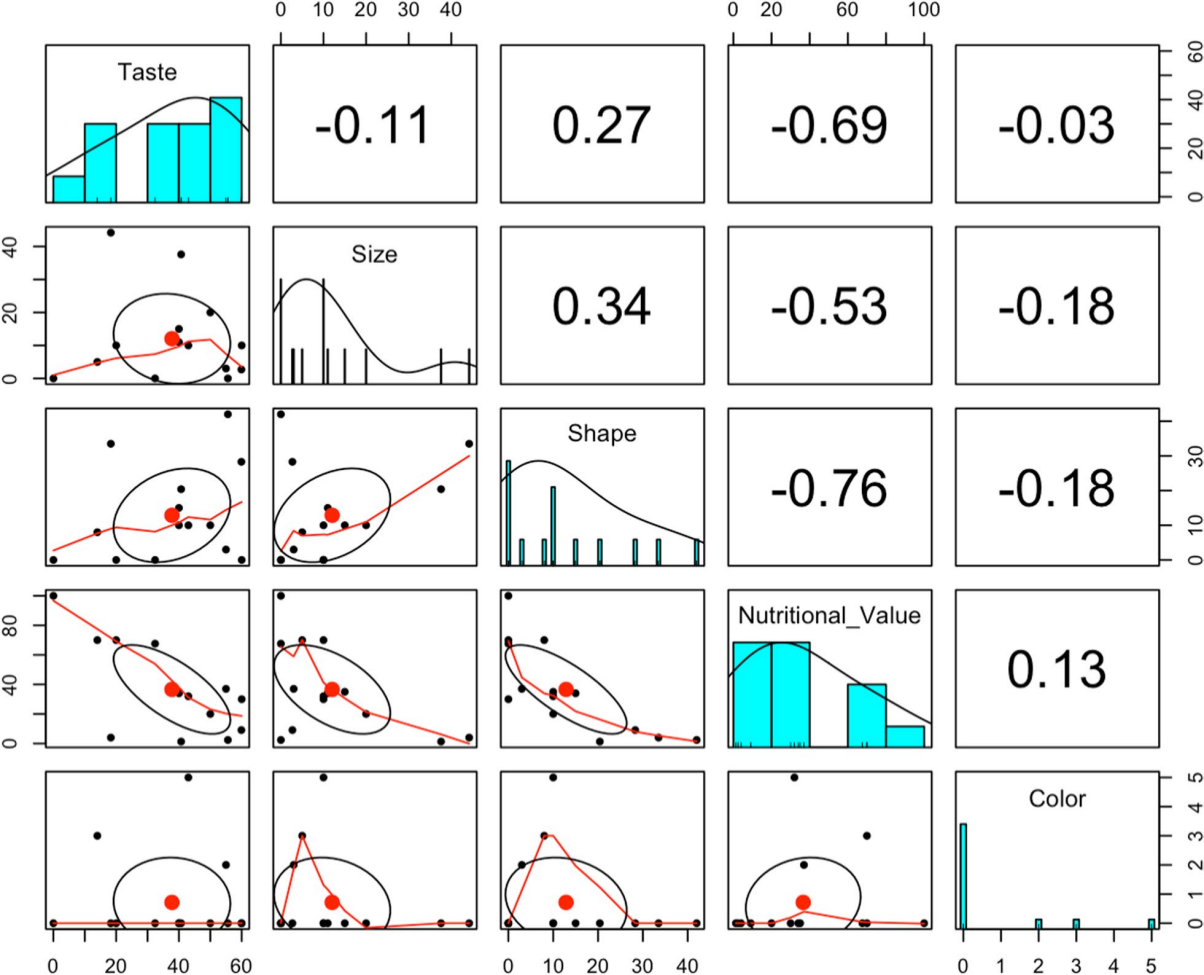
Scatter plot of matrices (SPLOM), histograms, and Pearson correlations between consumer preference factors

A scatter plot of matrices (SPLOM), histograms, and Pearson correlations between preference factors show a negative correlation between preference based on taste (r=-0.69), size (r = −0.53), and shape (r = −0.76), and preference based on nutritional value as depicted in Fig. 8.

### Seasonal availability of inventoried edible insects

Recorded information on the seasonal availability of inventoried edible insects in Kalehe and Idjwi territories showed that some are available throughout the year, while others are only available for 9 or even 4 months (Table 4). Like *A. mellifera*, *L. migratoria* is available throughout the year in both territories except that the latter is less available for 7 months in Kahele and 6 months in Idjwi. Some species are available for 9 months (*R. differens* in Kalehe, *G. africana* and *I. oyemensis* in Idjwi), others for 8 months (*I. oyemensis* in Kalehe, *M. subhyalinus* and *R. differens* in Idjwi), 7 months (*G. trivittata* and *R. phoenicis*), while *Vespula spp*. is only available during 4 months, of which 2 months of more availability and 2 months of less availability. Most of them are available in the rainy season, which runs from September to May in Kalehe and from September to April in Idjwi. Only species such as *A. mellifera* and *L. migratoria* are available in the dry season (June to August) in Kalehe, but the latter is not more available in this season. In contrast to Kalehe, four species of edible insects (*L. migratoria*, *A. mellifera*, *G. trivittata*, and *Vespula spp*) are available in Idjwi during the dry season.

**Table 4:** Seasonal availability for various consumed edible insects

Insect species	Rainy season					Dry season			Rainy season					
	Jan	Feb	Mar	Apr	May	Jun	Jul	Aug	Sep	Oct	Nov	Dec	TMA	TLA
Kalehe														
*I. oyemensis*	+	+	+	−	−	0	0	0	0	−	+	+	5	3
*L. migratoria*	+	−	−	−	−	−	−	−	+	+	+	+	5	7
*A. mellifera*	+	+	+	+	+	+	+	+	+	+	+	+	12	0
*M. subhyalinus*	+	+	+	−	−	0	0	0	0	−	+	+	5	3
*R. differens*	+	+	+	+	−	0	0	0	−	+	+	+	7	2
*G. africana*	+	+	+	+	+	0	0	0	+	+	+	+	9	0
Idjwi														
Idjwi														
*I. oyemensis*	+	+	+	+	−	0	0	0	−	+	+	+	7	2
*L. migratoria*	+	+	−	−	−	−	−	−	+	+	+	+	6	6
*A. mellifera*	+	+	+	+	+	+	+	+	+	+	+	+	12	0
*M. subhyalinus*	+	+	+	−	0	0	0	0	−	+	+	+	6	2
*R. differens*	+	+	−	−	0	0	0	0	−	+	+	+	5	3
*G. trivittata*	−	−	0	0	−	+	+	+	−	0	0	0	3	4
*R. phoenicis*	−	−	+	+	−	0	0	0	0	0	+	+	4	3
*Vespula spp*	0	0	0	0	−	−	+	+	0	0	0	0	2	2

+: month of availability

−: month of less availability

0: month of no availability

*TMA* Total month of availability; *TLA* Total month of less availability

### Host plants for various inventoried edible insects

While some edible insects require plants to serve as food sources or habitats, others do not necessarily need a host plant to survive. Table 5 shows that *L. migratoria* and *R. differens* are associated with crops such as *Zea mays*, *Sorghum bicolor*, *Phaseolus vulgaris*, *Ipomoea batatas*, *Oryza sativa*, *Saccharum officinarum*, and *Arachis hypogaea* as a food source or habitat, which are also used as staple food for humans in Kalehe and Idjwi territories. On the other hand, *R. phoenicis* feed on *Elaeis guineensis* and *Raffia palm* which are used for food and economic purposes. *Apis mellifera* is a pollinator and plays a critical role. *Macrotermes subhyalinus* and *G. africana* do not necessarily require a host plant. Indicators of their presence vary from species to species. The *I. oyemensis* is noticed by caterpillar smell and consumption of leaves, while *L. migratoria* is noticed by whistling, *G. africana* by whistling and canals in the wet ground, and *R. phoenicis* is noticed by cracking noises in palm trunks and odor.

**Table 5:** Host plants for various consumed edible insects in Kalehe and Idjwi territories

Insect species	Host plants		
	Common name	Scientific name	Indicators of presence
Kalehe			
*I. oyemensis*	Umbrella tree, red mangrove	*Maesopsis eminii, Rhizophora mangle*	Caterpillar smells and leaves consumed
*L. migratoria*	Maize, millet, bean, sweet potato	*Zea mays, Sorghum bicolor, Phaseolus vulgari, Ipomoea batatas*	Whistling
*A. mellifera*	NA	NA	NA
*M. subhyalinus*	NA	NA	NA
*R. differens*	Maize, millet, grass, Guinea grass,	*Zea mays, Sorghum bicolor, Digitaria* sp*, Panicum maximum*	NA
*G. africana*	NA	NA	Whistling and canals in the wet ground
Idjwi			
*I. oyemensis*	Sapele Mahogany, red mangrove	*Entandrophragma cylindricum, Rhizophora mangle*	Caterpillar smells and leaves consumed
*L. migratoria*	Maize, sorghum, rice, sugarcane, groundnut, sweet potato	*Zea mays, Sorghum bicolor, Oryza sativa, Saccharum officinarum, Arachis hypogaea, Ipomoea batatas*	Whistling
*A. mellifera*	NA	NA	NA
*M. subhyalinus*	NA	NA	NA
*R. differens*	Maize, millet, grass, giant rat's tail grass	*Zea mays, Sorghum bicolor, Digitaria* sp*, Sporobolis pyramidalis*	NA
*G. trivittata*	Giant rat's tail grass, weeping lovegrass, jaragua grass	*Sporobolis pyramidalis, Eragostis curvula, Hyparrhenia rufa,*	NA
*R. phoenicis*	African oil palm, palm	*Elaeis guineensis, Raffia palm*	Cracking noises in palm trunks and odor
*Vespula spp.*	Hogweed	*Heracleum* sp	NA

*NA* Not applicable

### Harvesting techniques and processing methods of edible insects

Harvesting techniques and period as well as processing methods and preservation techniques depend on the edible insect species, local knowledge, and practices (Table 6). Generally, two main techniques are used for harvest, namely handpicking and light trapping. The handpicking technique is used for *I. oyemensis*, *L. migratoria*, *G. africana*, and *R. phoenicis*, while *A. mellifera* and *Vespula spp.* are harvested. Unlike those species which are either only handpicked or harvested, *R. differens* and *M. subhyalinus* (during and after the first rains) are both light-trapped and handpicked. The harvesting period can be anytime during the day for some species such as *I. oyemensis*, *R. phoenicis*, and *Vespula spp.* Others including *L. migratoria* and *R. differens* are handpicked in the morning hours before sunrise when they are still weak and cannot fly. The eggs, larvae, and pupae of *A. mellifera* are preferably harvested at night.

**Table 6:** Harvesting techniques and processing methods for various consumed edible insects in selected territories

Insect species	Harvesting techniques	Harvesting period	Processing methods	Preservation techniques
Kalehe				
*I. oyemensis*	Direct handpicking or after shaking trees and branches.	Any time, morning and evening hours preferably	Boiled, fried, and stewed sometimes	Drying
*L. migratoria*	Handpicking	Morning time	De-winged, roasted, or dry-fried	Drying
*A. mellifera*	Harvesting honeycomb from the hive followed by honey extraction	Preferably at night	Boiled	None
*M. subhyalinus*	Handpicking and Light trapping near a container.	During and after the first rains	De-winged, dry-fried, and eaten raw sometimes	Drying
*R. differens*	The light trapping technique and handpicking during the swarming season	In the dark for light trapping and morning for handpicking	De-winged, dry-fried, or roasted	Dry-fried
*G. africana*	Handpicking after digging	After the dark preferably, following their small holes	De-winged, roasted, and fried	Drying
Idjwi				
*I. oyemensis*	Direct handpicking after their signs of presence are detected.	Any time, preferably morning and evening hours	Boiled, roasted, and stewed	Drying
*L. migratoria*	Handpicking	Morning time	De-winged, roasted, or dry-fried	Drying
*A. mellifera*	Harvesting honeycomb from the hive followed by honey extraction	At night preferably	Boiled	None
*M. subhyalinus*	Light trapping near a container filled with water	During and after the first rains	De-winged, roasted, and dry-fried	Drying
*R. differens*	Light trapping and handpicking during the swarming season.	Soon after the dark (light trapping) and morning (handpicking)	De-winged, dry-fried	Dry-fried
*R. phoenicis*	Handpicking after signs of their presence are detected.	Any time	Boiled, dry-fried, fried, or stewed sometimes	Drying
*Vespula spp.*	Harvesting	Any time	Boiled	None

Processing methods and preservation techniques vary from species to species and purposes whether it is for direct consumption or preservation for further uses or market. Some species are boiled (*I. oyemensis*, and the larvae of *A. mellifera* and *Vespula spp*), fried (*I. oyemensis* and *G. africana*), or dry-fried (*L. migratoria*, *M. subhyalinus*, *R. differens*, and *R. phoenicis*), stewed (*I. oyemensis* and *R. phoenicis*), roasted (*L. migratoria*, *R. differens*, *G. africana*, *I. oyemensis*, and *M. subhylanus*), and sometimes eaten raw (*M. subhyalinus*). All edible insects with wing (*L. migratoria*, *M. subhylanus*, *R. differens*, and *G. africana*) are first de-winged before being processed. If there is enough to preserve, most of the edible insects are usually dried (*I. oyemensis*, *L. migratoria*, *M. subhyalinus*, *G. africana*, and *R. phoenicis*) or dry-fried (*R. differens*) except for the immature stages of *A. mellifera* and *Vespula spp*. All edible insects with wings (*L. migratoria*, *M. subhylanus*, *R. differens*, and *G. africana*) are first de-winged before being processed.

## Discussion

A total of nine edible insects were identified as a food source in Kalehe and Idjwi territories belonging to nine families and five orders, confirming the wide edible insects’ diversity in the Democratic Republic of Congo in general and South-Kivu Province in particular. This is largely in agreement with Ishara and collaborators [11], who conducted a similar study in Fizi, Kabare, Mwenga, and Walungu territories, reporting a total of 23 edible insects used as a food source belonging to the same orders but nine families. Similarly, Bomolo and collaborators [39] reported eleven edible insects belonging to four families that are consumed as a food source in Haut-Katanga Province, confirming the idea that the Democratic Republic of Congo is one of the host-spots of edible insects in Africa, although this richness in terms of edible insect biodiversity remains poorly documented. Additionally, the revealed wide biodiversity of edible insects observed in the study area may be related to the agroecological conditions of the area resulting in more availability of host plants serving as their source of food and habitat for most edible insects.

The recorded edible insects are generally consumed as larvae, adults, or both larvae and nymph (*I. oyemensis*), except *A. millifera* which is consumed as egg, larvae, and pupae. Our findings corroborate those of Akullo and collaborators [12] who found that termites (*Macrotermes spp.*) and grasshoppers (*Cyrtacanthacris aeruginosa*, *Zonocerus variegatus*) are consumed as adults, and *A. mellifera* as egg, larvae, and pupae in Lango subregion, Northern Uganda. Additionally, Kelemu and collaborators [14] reported Lepidoptera (caterpillars) and Hymenoptera (*A. mellifera*) to be consumed as adults and larvae, while the orders of Orthoptera, Blattodea, and Hemiptera are mainly consumed as adults. Note that the developmental stage of edible insects does not have significant variations on their nutritional values except for protein digestibility, fat content, and lipid quality [40].

Most of these edible insects have local names generally derived from the dialects Kihavu and Kitemba in Kalehe, and Kihavu in Idjwi referring to physical characteristics or uses. This is consistent with several studies that also reported local names according to local dialects alluding to physical characteristics or uses [11, 12, 41].

It was noted that inventoried edible insects are differentially preferred, with *R. differens* and *M. subhyalinus* being the most preferred regardless of the territory. These edible insects are the most familiar in the study area confirming the fact that edible insects’ preference is mainly influenced, on the one hand, by their familiarity [9, 17], culture [42], palatability [17], and availability and, on the other hand, by local knowledge and processing [15]. A study in the Netherlands [18] reported that people who had eaten insects in the past showed significantly more positive attitudes toward entomophagy than people who had not eaten them and were more likely to eat them again. The preference was found to be influenced by several factors, including taste, shape, size, nutritional value, and color, supporting the findings of Van Huis [9] and Riggi and collaborators [15] stating that insect consumption depends not only on sensory characteristics [9] and nutritional value [15, 19], but also on customs, ethnic preferences, prohibitions [20], and medicinal properties [43]. Ghosh and others [44] explored what governs selection as well as acceptance of edible insect species and found that traditions obviously play a role, highlighting that superstition and taboos will have been major factors. They further added that climatic and ecological characteristics that influence the locally available food insect spectrum and looks, taste, and feel of an insect are further features also come into play.

The seasonal availability of inventoried edible insects in Kalehe and Idjwi territories depends on the species of insect. This is similar to the findings of Ebenebe and collaborators [21], who noted that alates termites, crickets, caterpillars, *A. domesticus*, *G. africana*, and the greenish beetle were more available during the rainy season in Nigeria. Similarly, peak numbers of edible beetles occur from June to September, while Odonata and Orthoptera were most abundant from September to October [17]. This agrees with our findings showing that most edible insects in the study area were more available in the rainy seasons. Since most edible insects are wild collected in the Kalehe and Idjwi territories, having agroecological conditions favoring the growth of host plants in this study area [45], could be associated with the wide biodiversity observed in Kalehe and Idjwi. The high population density observed in Idjwi (1032.3hab.km^-2^) followed a strong pressure on the environment and expansion of agricultural activities in detriment of forests is very crucial for species that have forest species as hosts [46].

While some edible insects require host plants to survive, others do not necessarily need host plants to survive. Plus, some edible insect species feed on staple crops, which are also source of food and income in Kalehe and Idjwi territories. Our results coincide with those of Ebenebe and collaborators [21] who also reported that edible insects use various host plants including plants used as source of food and income in Nigeria. In Cameroon, eighteen plants were identified as host plants for edible insects, eleven of which are restricted to natural forest habitats, including *Entandrophragma cylindricum* and *Baillonella toxisperma*, and others domesticated and grown in home gardens [22]. Other studies [47] have also noted that among host plants are those that produce valuable fruits and from which edible oil is extracted, which are a major source of income.

The detection of edible insects varies from species to species, and as Meutchieye and collaborators [24] noted, cracking noises in palm trunks and caterpillar odors as indicators of *R. phoenicis* confirm our findings reporting that *I. oyemensis* is noticed by its odor and its consumption of leaves, while *L. migratoria* is noticed by whistling, *G. africana* by whistling and canals in the wet ground, and *R. phoenicis* is noticed by cracking noises in palm trunks and odor.

We found that three main techniques are generally used for harvesting in Kalehe and Idjwi, namely handpicking, harvesting, and light trapping, supporting the findings of Meutchieye and collaborators [24] and Ebenebe and collaborators [21] who pointed out that handpicking is one of the main techniques for harvesting crickets, rhinoceros beetle, grasshopper, African palm weevil, and caterpillars, adding in agreement with our findings that light trapping is used to harvest alates termites, green locusts, house locust, and mole locust. Light trapping is used for swarming *R. differens*, while handpicking is done very early in the morning between 6:00 and 7:00 for non-swarming *R. differens*, when they cannot fly [48].

Like harvesting techniques and periods, processing methods and preservation techniques also vary from species to species based on purpose, whether for direct consumption or preservation for later uses or sales. Some species are boiled, fried, dry-fried, stewed, roasted, and sometimes eaten raw (*M. subhyalinus*). Our findings are consistent with those of Ayieko and collaborators [25], and Chung [26] who reported that edible insects are sun-dried, baked, and steamed. Ebenebe and collaborators [21] highlighted as we do that salt-roasting is one of the techniques used to process termites, crickets, rhinoceros beetle, grasshopper, and cricket. For preservation, most edible insects are usually dried or dry-fried, except *A. mellifera* and *Vespula spp*, as drying including roasting, frying, and solar drying which is the most used preservation technique to increase the shelf life of edible insects [27].

## Conclusion and recommendations

The nine edible insects identified as a food source in Kalehe and Idjwi serve as evidence of entomophagous practices in the area. The recorded edible insects belong to nine families and five orders and are consumed as larvae, adults, or as eggs and pupae, with *R. differens* and *M. subhyalinus* being the most preferred. Their seasonal availability varies from one species to another. Most of them are available in the rainy seasons, perhaps because of the abundance of food and host plants at that time. Among the host plants used, some are used as source of food for humans and source of income in the Kalehe and Idjwi territories.

Generally, two main harvesting techniques are used, namely handpicking and light trapping, depending on the species, local knowledge, and practices. Processing methods and preservation techniques also vary from species to species, whether it is for direct consumption or preservation for later use or market. Most edible insects are usually dried or dry-fried for preservation, except for immature of *A. mellifera* and *Vespula* spp.

Similar country-wide studies focusing on the insects and their nutritional as well as environmental advantages over conventional livestock are to be encouraged. Moreover, in view of these insects’ abundance, their rearing is both economic and sustainable and for these reasons ought to be supported in the South-Kivu Province.

## Data Availability

Most generated data during this study are included in this manuscript. More information is available on reasonable request.

## Code availability

All RStudio codes are open source and available primarily at https://www.rstudio.com.
